# Supplementary material for: Severe SARS-CoV-2 infection in humans is defined by a shift in the serum lipidome resulting in dysregulation of eicosanoid immune mediators
Source: Res Sq. 2020 Jul 22:rs.3.rs-42999. Preprint. [Version 1] doi: 10.21203/rs.3.rs-42999/v1 (PMC7386513; doi:10.21203/rs.3.rs-42999/v1)

Severe SARS-CoV-2 infection in humans is defined by a shift in the serum lipidome resulting in dysregulation of eicosanoid immune mediators

Benjamin Schwarz, Lokesh Sharma, Lydia Roberts, Xiaohua Peng, Santos Bermejo, Ian Leighton, Arnau Casanovas Massana, Shelli Farhadian, Albert I. Ko, Yale IMPACT Team, Charles S. Dela Cruz, Catharine M. Bosio

Supplementary Material

Supplementary Figure Captions

Supplemental Figure 1. Systemic changes in COVID-19 patient serum amino acid pools, lactate directed glycolysis and the xanthine oxidase stress response. (a) Unsupervised principle component analysis (PCA) of the autoscaled aqueous metabolite dataset and **(b)** corresponding loading plot of feature contribution to each axis of variance. **(c)** Supervised partial least square discriminant analysis (PLSDA) of the healthy, moderate and severe disease groups and **(d)** corresponding feature loading plot. Univariate analysis of aqueous metabolites by unpaired t-test comparing moderate disease to healthy **(e)**, severe disease to healthy **(f)** or severe to moderate disease **(g)**. Cutoff lines indicate a positive or negative 2-fold change and a p-value of 0.05. **(h & i)** Heatmaps of the autoscaled mean of each group for significantly varied molecules ($p<0.05$) in **(h)** either of the disease vs healthy comparisons or **(i)** the severe to moderate comparison.

Supplemental Figure 2. Multiple lipid classes contribute to the multivariate and univariate separation of disease cohorts. (a) Unsupervised principle component analysis (PCA) of pareto scaled negative ionization lipid dataset and **(b)** corresponding feature loading plot. **(c)** Unsupervised PCA of pareto scaled positive ionization lipid dataset and **(d)** corresponding feature loading plot. **(e-p)** Comparison of

moderate to healthy **(e,h,k,n)**, severe to healthy **(f,i,l,o)** and severe to moderate **(g,j,m,p)** by unpaired t-test. **(e-g)** Neutral lipids species including monoacylglycerol, diacylglycerol, and triacylglycerol are overlaid in red. **(h-j)** Lyso-phospholipids are overlaid including lysophosphatidylcholine (Lyso-PC, blue), lysophosphatidylethanolamine (Lyso-PE, orange) and lysophosphatidylserine (Lyso-PS, purple). **(k-m)** Cholesterol-esters (Chol-Est) are overlaid in red. **(n-p)** Sphingolipids are overlaid including sphingomyelin (SM, blue), ceramide (Cer, orange), dihydroceramide (DCer, purple) and hexosylceramides (HCer, red). For (e-p) cutoff lines indicate a positive or negative 2-fold change and a p-value of 0.05.

Supplemental Figure 3. AA series LM spectra confirmations. Triggered spectra for the AA-derived LMs collected from patient samples within the cohort to confirm the identity of each lipid species. All molecules were compared to available database of authentic spectra collected on a comparable instrument (http://serhanlab.bwh.harvard.edu/wp-content/uploads/2019/05/UPDATED-2019-Spectra-Book.pdf)[1] or, for the DiHETrE series, compared to predicted spectra from Human Metabolome Database[2]. Peaks that are part of the known reference spectra or predicted spectra are shown in red.

Supplemental Figure 4. EPA series spectra confirmations. Triggered spectra for the EPA-derived LMs collected from patient samples within the cohort to confirm the identity of each lipid species. All molecules were compared to available database of authentic spectra collected on a comparable instrument(http://serhanlab.bwh.harvard.edu/wp-content/uploads/2019/05/UPDATED-2019-Spectra-Book.pdf)[1]. Peaks that are part of the known reference spectra shown in red.

Supplemental Figure 5. DHA series spectra confirmations. Triggered spectra for the DHA-derived LMs collected from patient samples within the cohort to confirm the identity of each lipid species. All molecules were compared to available database of authentic spectra collected on a comparable

instrument (http://serhanlab.bwh.harvard.edu/wp-content/uploads/2019/05/UPDATED-2019-Spectra-Book.pdf)[1]. Peaks that are part of the known reference spectra shown in red.

Supplemental Figure 6. Expression of ALOX and Cytochrome p450 genes in healthy or COVID human PBMCs. DotPlot visualization of **(a)** *ALOX5*, *ALOX12*, and *ALOX15* or **(b)** *CYP4F2*, *CYP4A11*, *CYP2J2*, *CYP2C9*, and *CYP2C*8 gene expression in healthy (blue) or COVID (gray) PBMC cellular populations from single cell RNA-Seq data published in Wilk, et al *Nature Medicine* 2020.[3] The dot size indicates the percentage of cells within a certain population that express the indicated gene.

Supplemental Figure 7. Overlay of patient sex (blue- female, red- male) with unsupervised PCAs of the **(a)** autoscaled aqueous metabolite dataset, **(b)** autoscaled combined lipid mediator and cytokine dataset, **(c)** pareto scaled negative ionization lipid dataset, and **(d)** pareto scaled positive ionization lipid dataset. The percentage of female patients was 47.4% in the healthy cohort, 37.5% in the moderate COVID group, and 39.1% in the severe COVID group.

Supplemental Figure 8. Overlay of patient age (blue- < 65 years old, red- > 65 years old) with unsupervised PCAs of the **(a)** autoscaled aqueous metabolite dataset, **(b)** autoscaled combined lipid mediator and cytokine dataset, **(c)** pareto scaled negative ionization lipid dataset, and **(d)** pareto scaled positive ionization lipid dataset. The percentage of patients < 65 years old was 89.5% in the healthy cohort, 62.5% in the moderate COVID group, and 34.8% in the severe COVID group.

Supplemental Figure 9. Overlay of antiviral treatment (gray- healthy controls, blue- none, red- Azatanaivir, purple- Remdesivir) with unsupervised PCAs of the **(a)** autoscaled aqueous metabolite dataset, **(b)** autoscaled combined lipid mediator and cytokine dataset, **(c)** pareto scaled negative

ionization lipid dataset, and **(d)** pareto scaled positive ionization lipid dataset. The percentage of patients treated with Azatanaivir was 66.7% in the moderate COVID group and 69.6% in the severe COVID group. The percentage of patients treated with Remdesivir was 12.5% in the moderate COVID group and 17.4% in the severe COVID group.

Supplemental Figure 10. Overlay of Tocilizumab treatment (gray- healthy controls, blue- none, red- Tocilizumab) with unsupervised PCAs of the **(a)** autoscaled aqueous metabolite dataset, **(b)** autoscaled combined lipid mediator and cytokine dataset, **(c)** pareto scaled negative ionization lipid dataset, and **(d)** pareto scaled positive ionization lipid dataset. The percentage of patients treated with Tocilizumab was 54.2% in the moderate COVID group and 87.0% in the severe COVID group.

Supplemental Figure 11. Overlay of hydroxychloroquine (HCQ) treatment (gray- healthy controls, blue- none, red- HCQ) with unsupervised PCAs of the **(a)** autoscaled aqueous metabolite dataset, **(b)** autoscaled combined lipid mediator and cytokine dataset, **(c)** pareto scaled negative ionization lipid dataset, and **(d)** pareto scaled positive ionization lipid dataset. The percentage of patients treated with HCQ was 75% in the moderate COVID group and 100% in the severe COVID group.

Supplemental Figure 12. Overlay of patient BMI (gray- healthy controls, blue- BMI < 30, red- BMI > 30) with unsupervised PCAs of the **(a)** autoscaled aqueous metabolite dataset, **(b)** autoscaled combined lipid mediator and cytokine dataset, **(c)** pareto scaled negative ionization lipid dataset, and **(d)** pareto scaled positive ionization lipid dataset. The percentage of patients with a BMI < 30 was 54.2% in the moderate COVID group and 39.1% in the severe COVID group.

Supplemental Figure 13. Overlay of patient diabetic status (gray- healthy controls, blue- Not diabetic, red- Diabetic) with unsupervised PCAs of the **(a)** autoscaled aqueous metabolite dataset, **(b)** autoscaled combined lipid mediator and cytokine dataset, **(c)** pareto scaled negative ionization lipid dataset, and **(d)** pareto scaled positive ionization lipid dataset. The percentage of patients with diabetes was 29.2% in the moderate COVID group and 34.8% in the severe COVID group.

Supplemental Figure 14. Overlay of patient heart disease status (gray- healthy controls, blue- no heart disease, red- heart disease) with unsupervised PCAs of the **(a)** autoscaled aqueous metabolite dataset, **(b)** autoscaled combined lipid mediator and cytokine dataset, **(c)** pareto scaled negative ionization lipid dataset, and **(d)** pareto scaled positive ionization lipid dataset. The percentage of patients with heart disease was 33.3% in the moderate COVID group and 52.2% in the severe COVID group.

Supplemental Figure 15. Overlay of patient survival (gray- healthy controls, blue- discharged, red- deceased) with unsupervised PCAs of the **(a)** autoscaled aqueous metabolite dataset, **(b)** autoscaled combined lipid mediator and cytokine dataset, **(c)** pareto scaled negative ionization lipid dataset, and **(d)** pareto scaled positive ionization lipid dataset. The percentage of patients that survived was 100% in the moderate COVID group and 56.5% in the severe COVID group.

Supplementary References

1. Ian Riley, P.C.N., Kathy Luong, Charles Serhan. CET&RI Spectra Book & Physical Properties of SPM and Eicosanoids 2019. (2019).
2. Wishart, D.S., *et al.* HMDB 4.0: the human metabolome database for 2018. *Nucleic acids research* **46**, D608-D617 (2018).
3. Wilk, A.J., *et al.* A single-cell atlas of the peripheral immune response in patients with severe COVID-19. *Nature Medicine*, 1-7 (2020).

Supplemental Figure 1

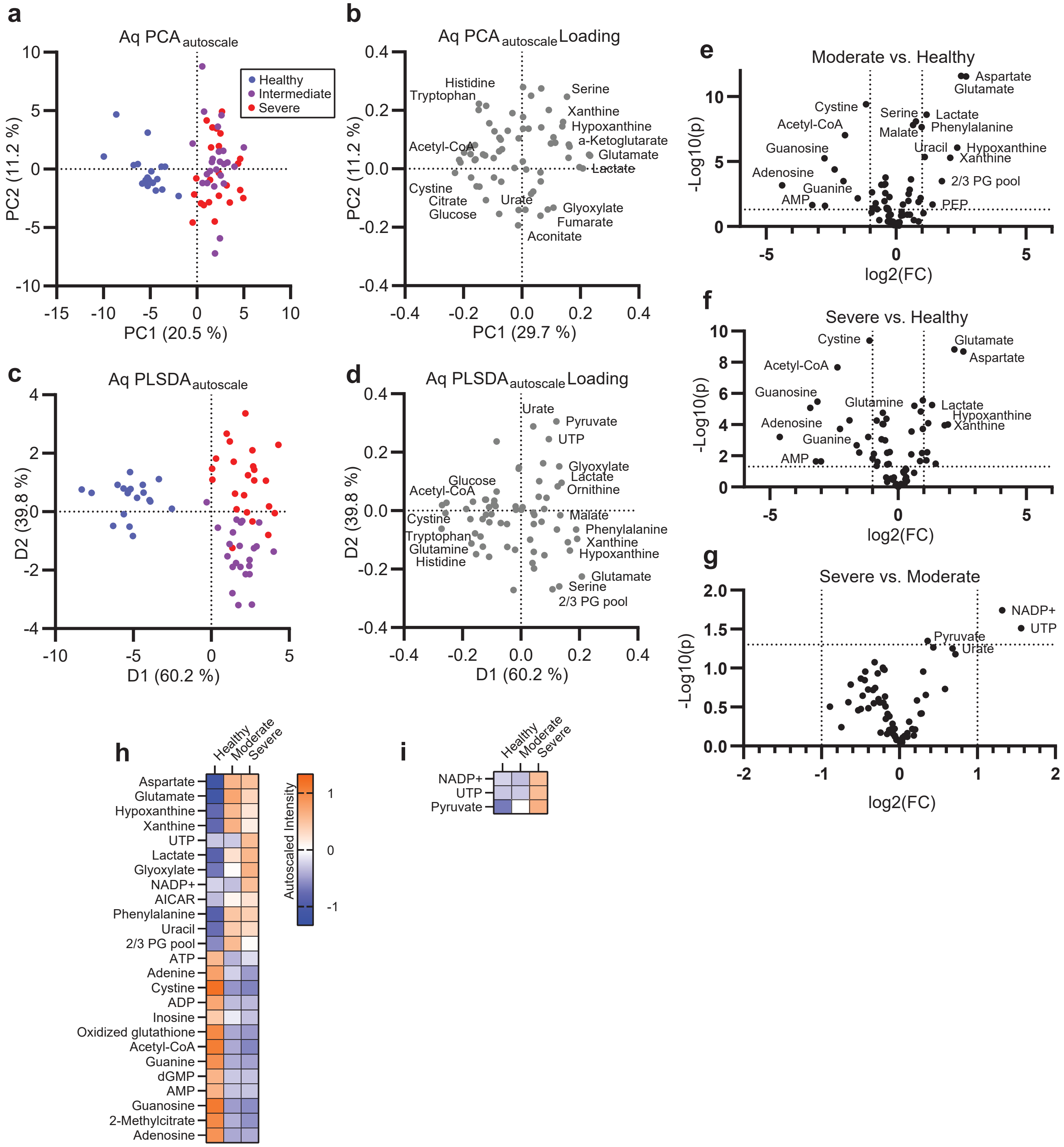

Supplemental Figure 2

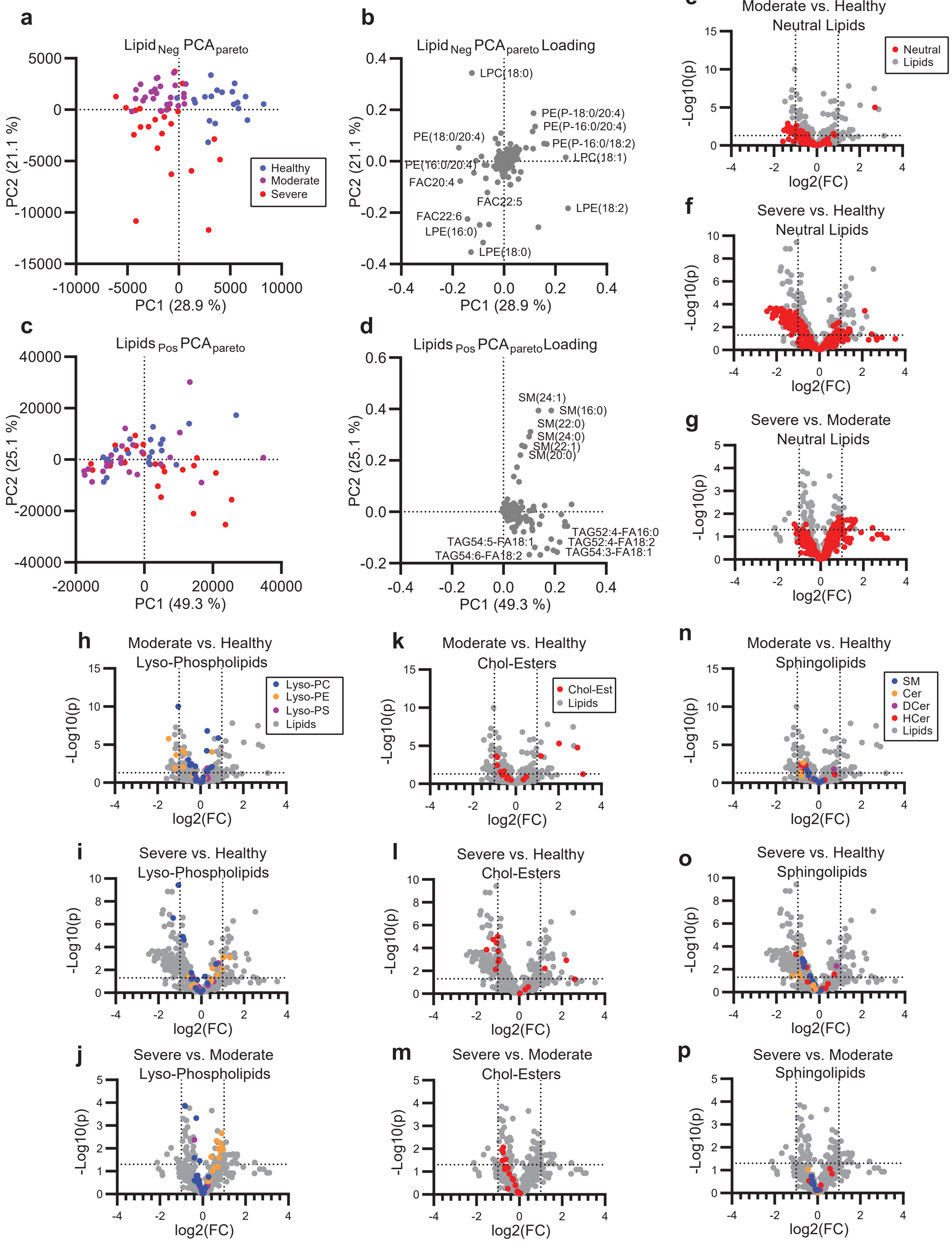

Supplemental Figure 3

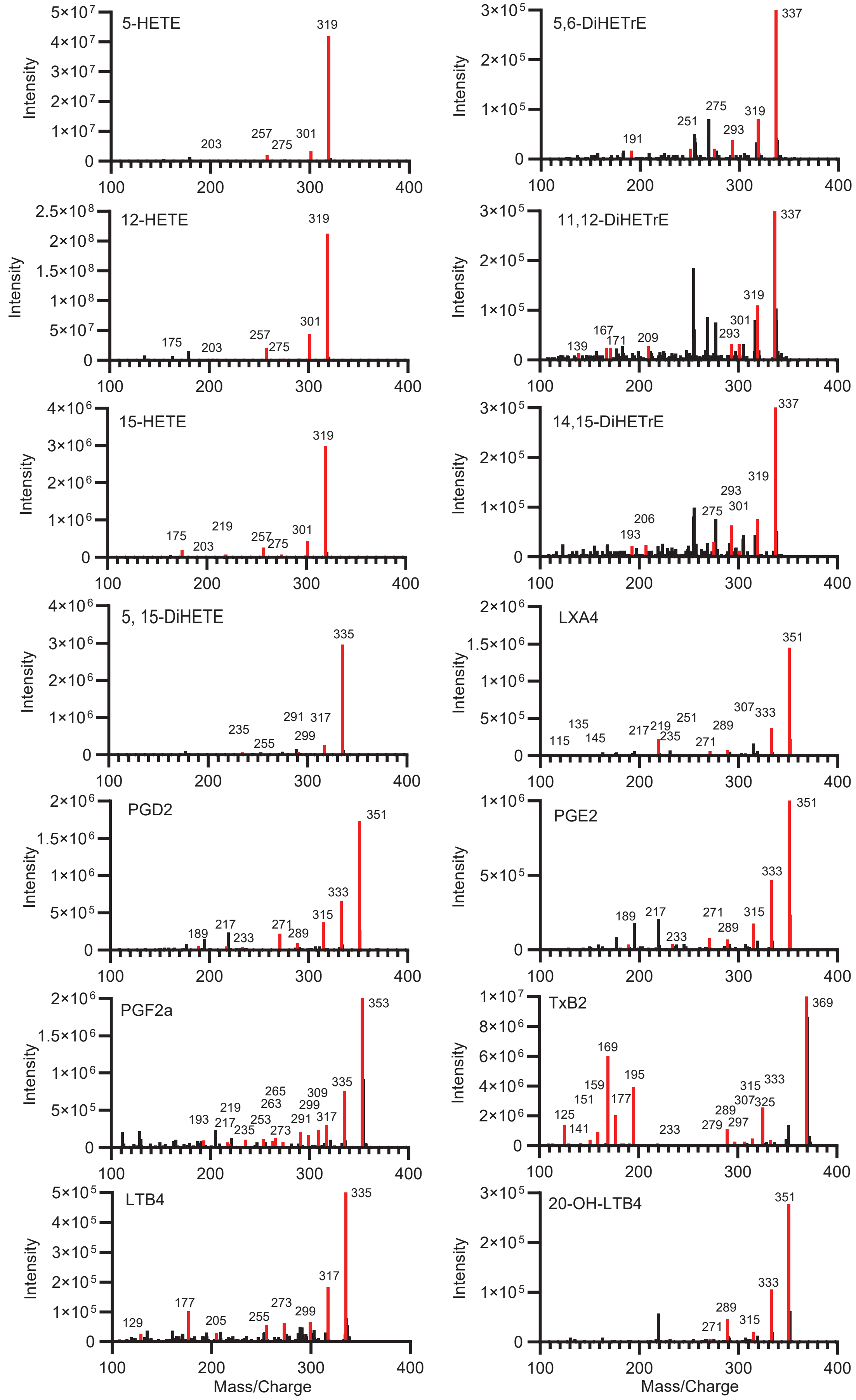

Supplemental Figure 4

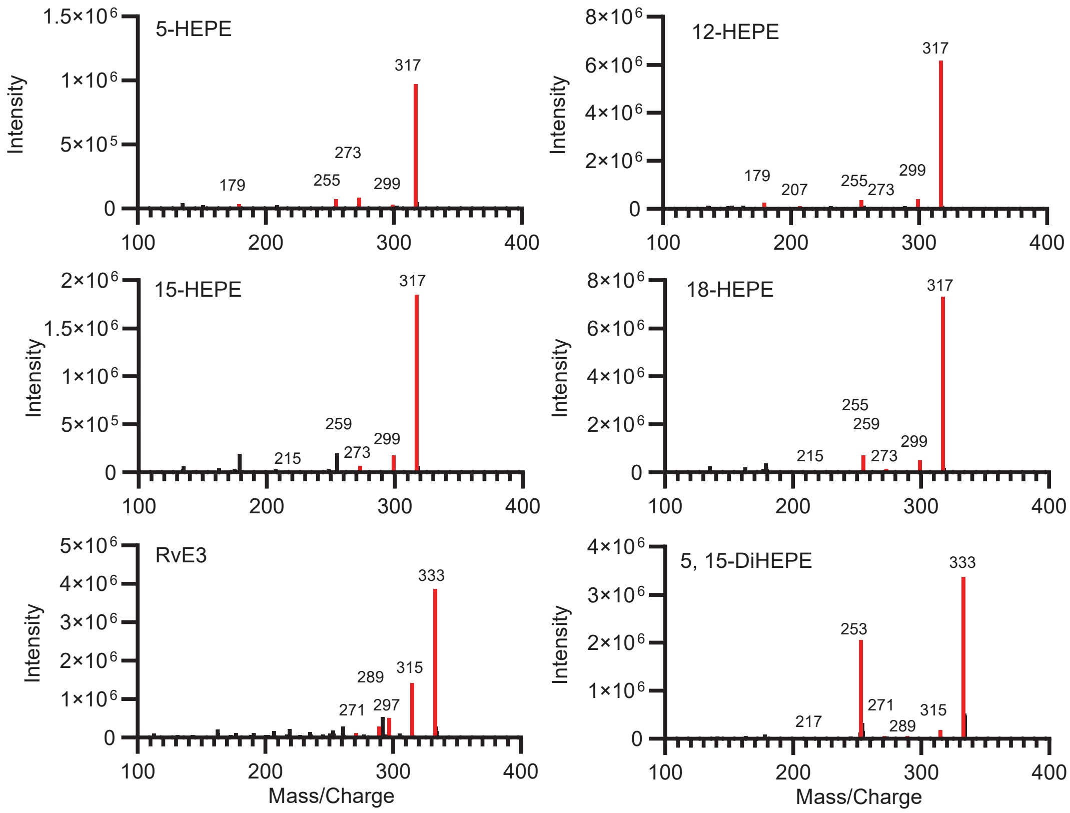

Supplemental Figure 5

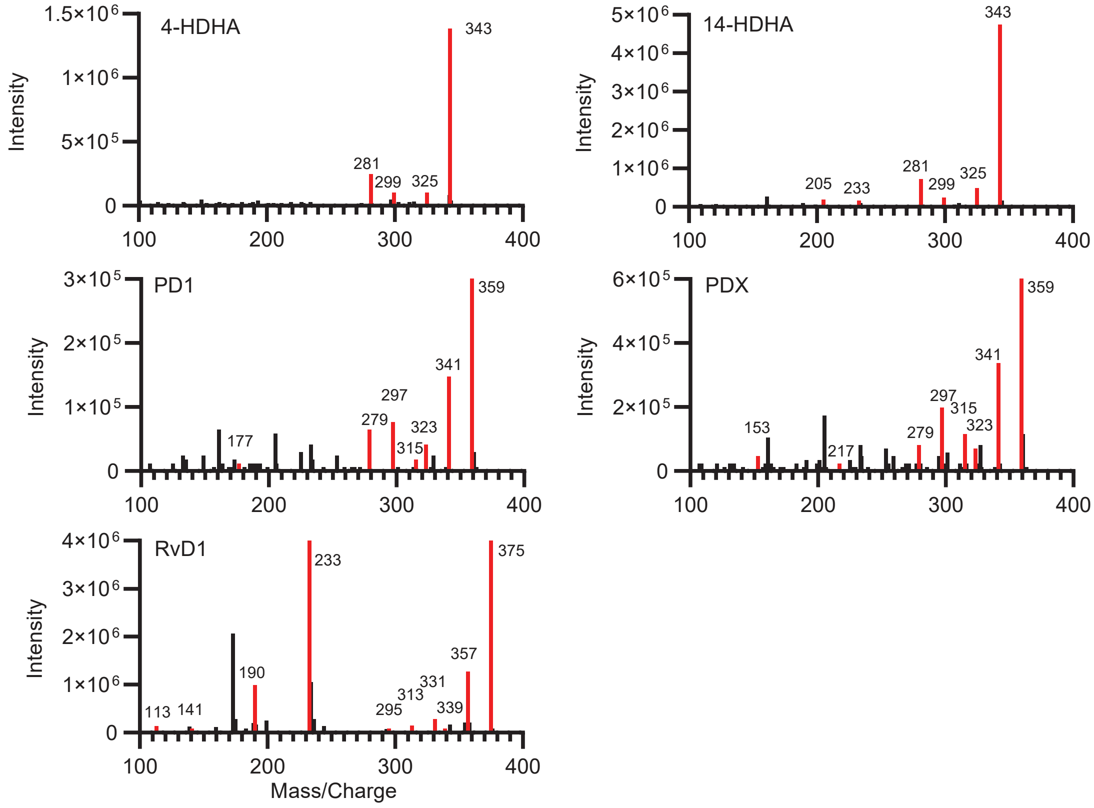

Supplemental Figure 6

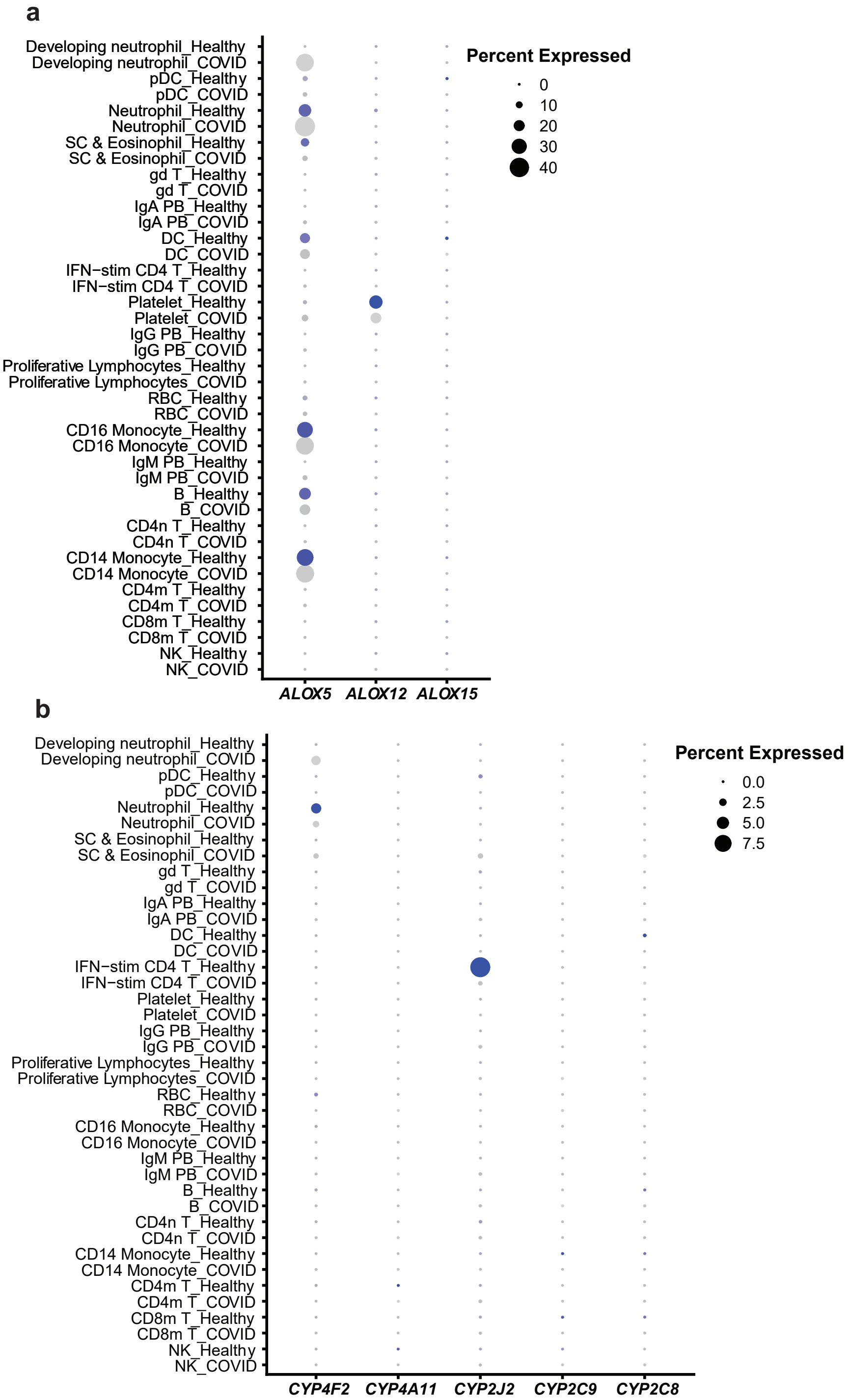

Supplemental Figure 7

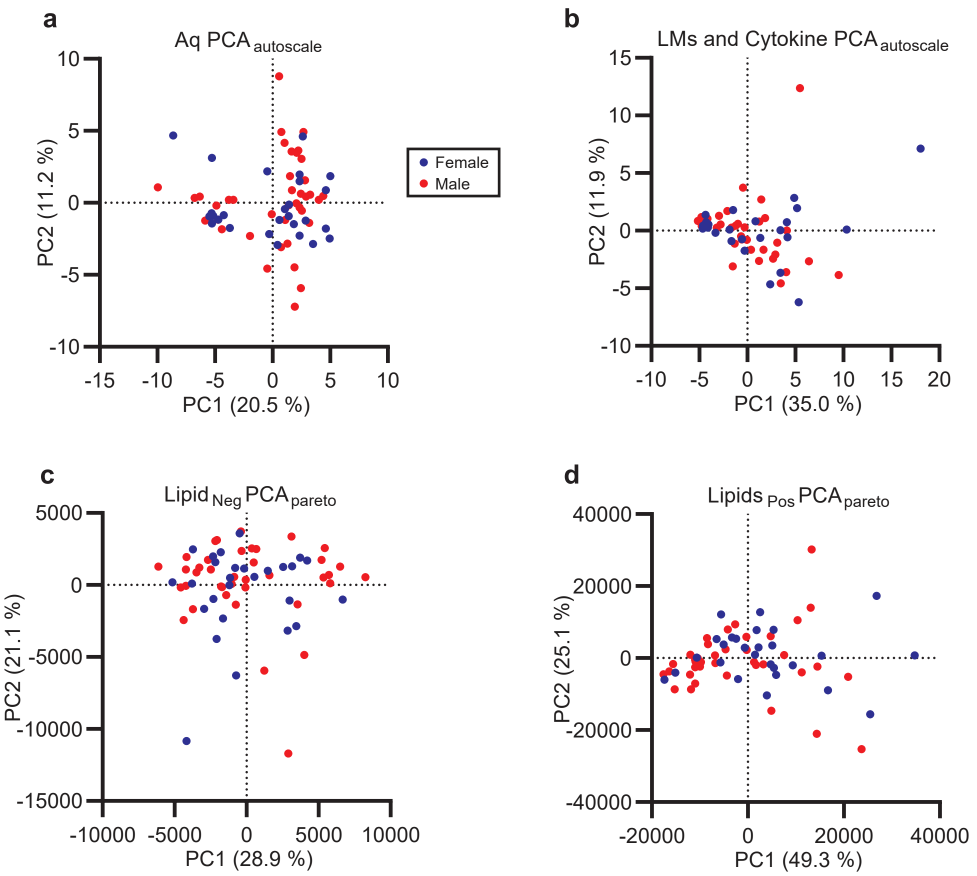

Supplemental Figure 8

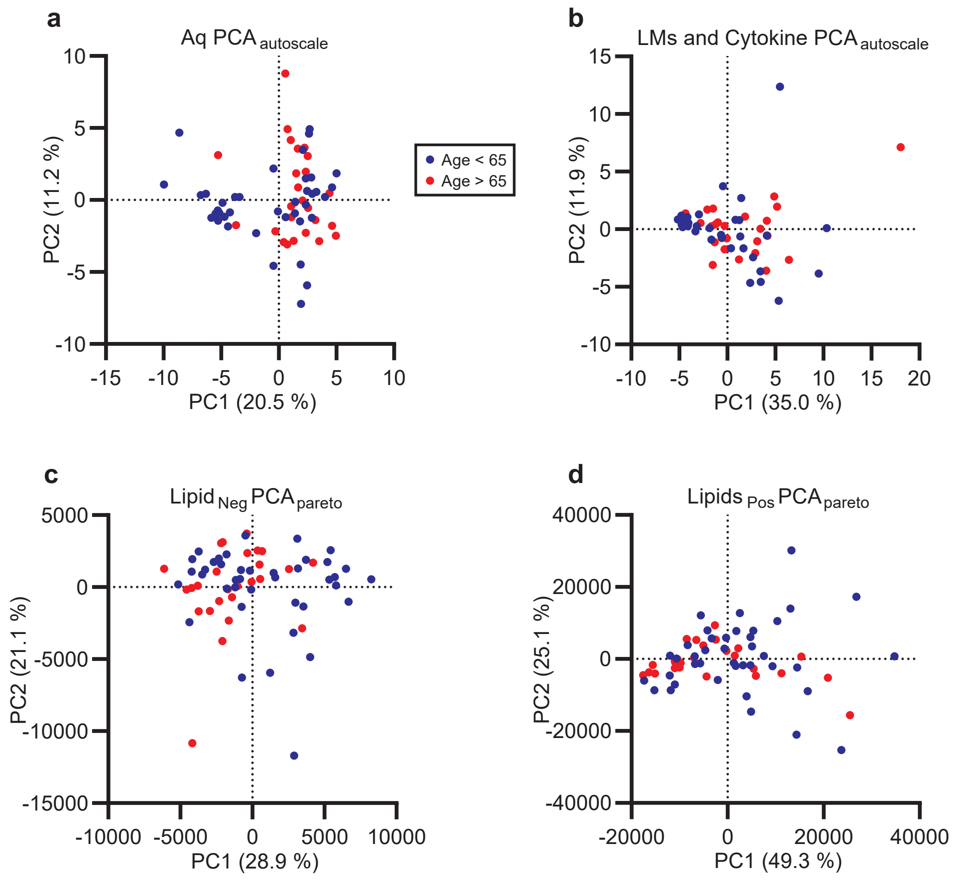

Supplemental Figure 9

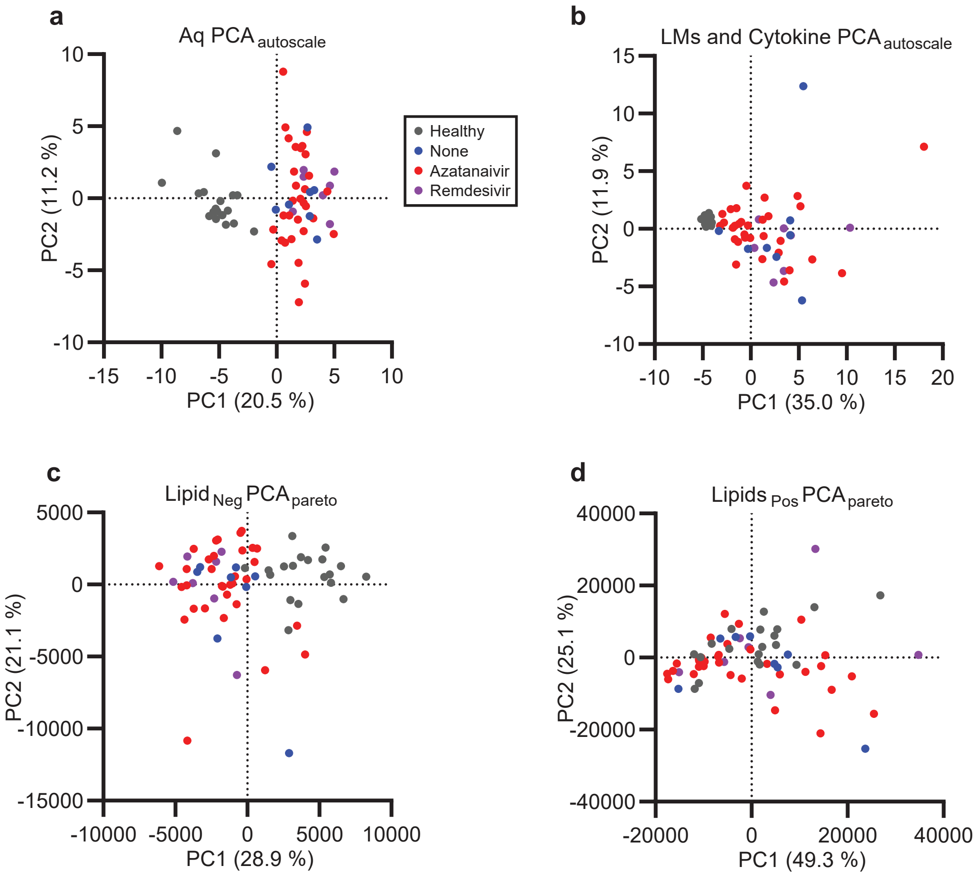

Supplemental Figure 10

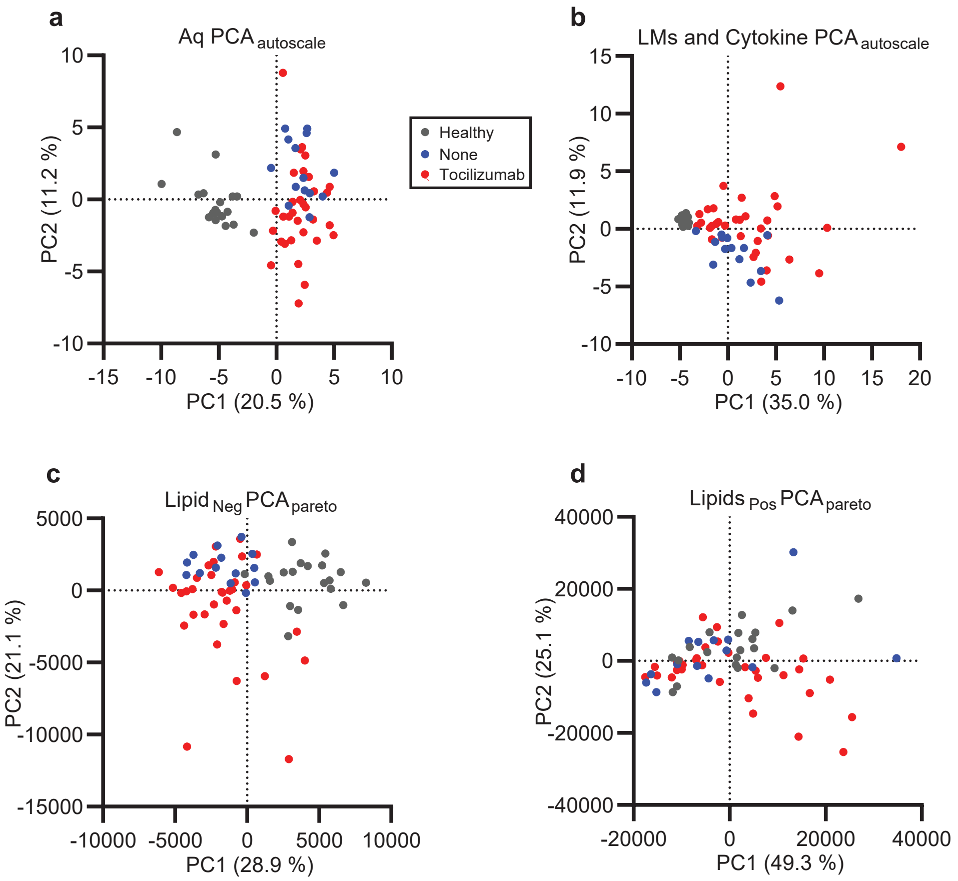

Supplemental Figure 11

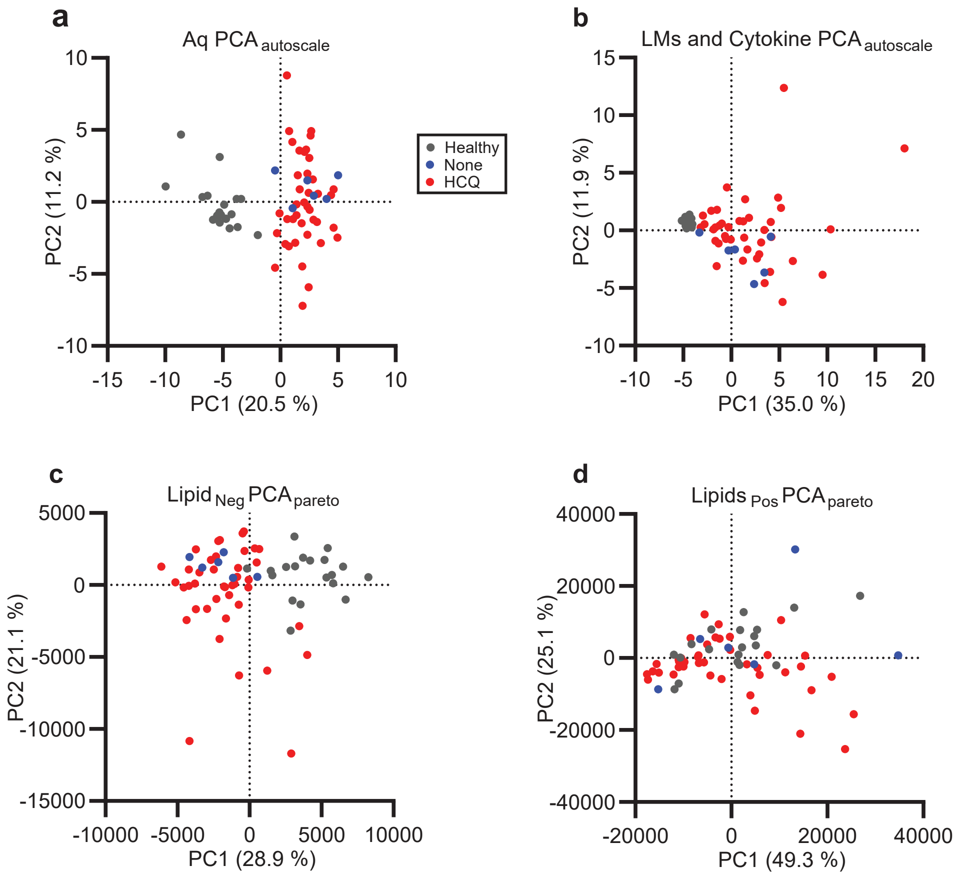

Supplemental Figure 12

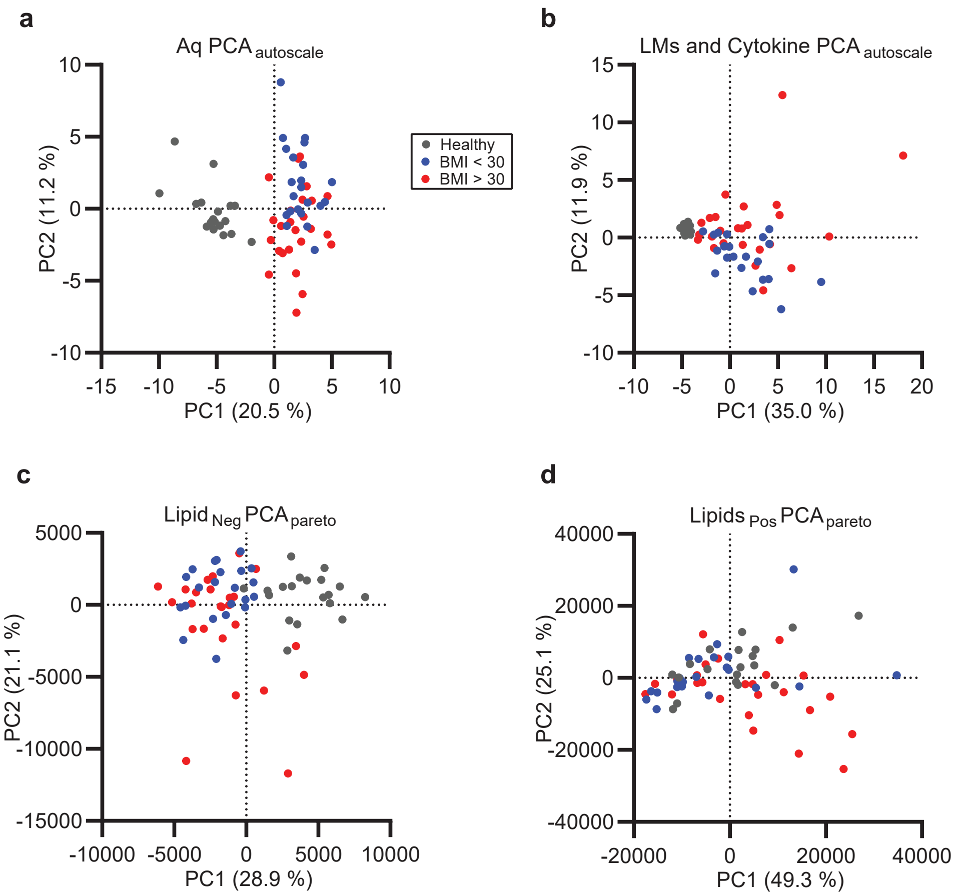

Supplemental Figure 13

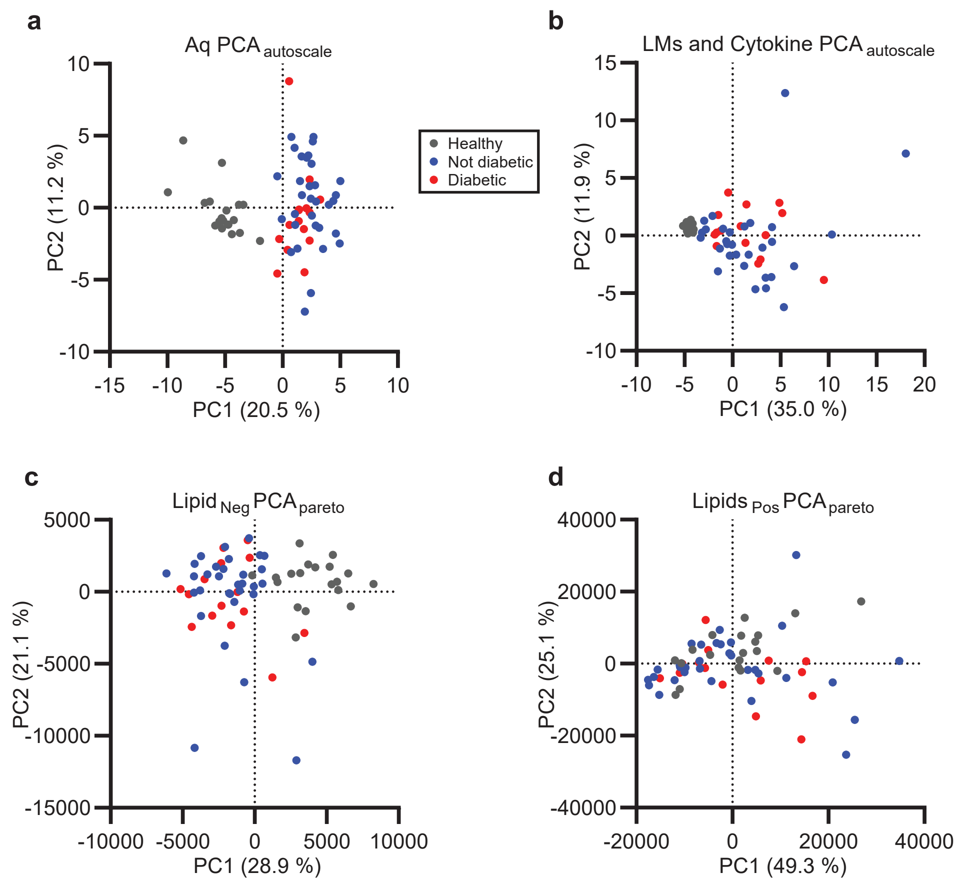

Supplemental Figure 14

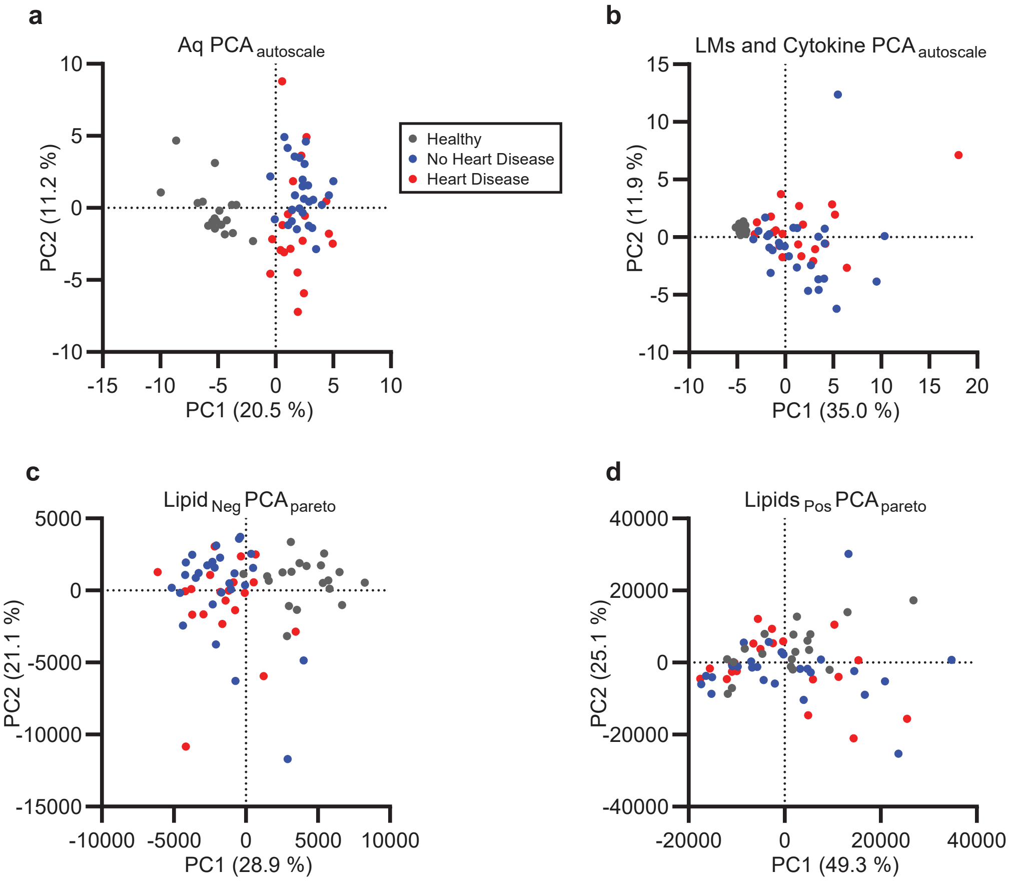

Supplemental Figure 15

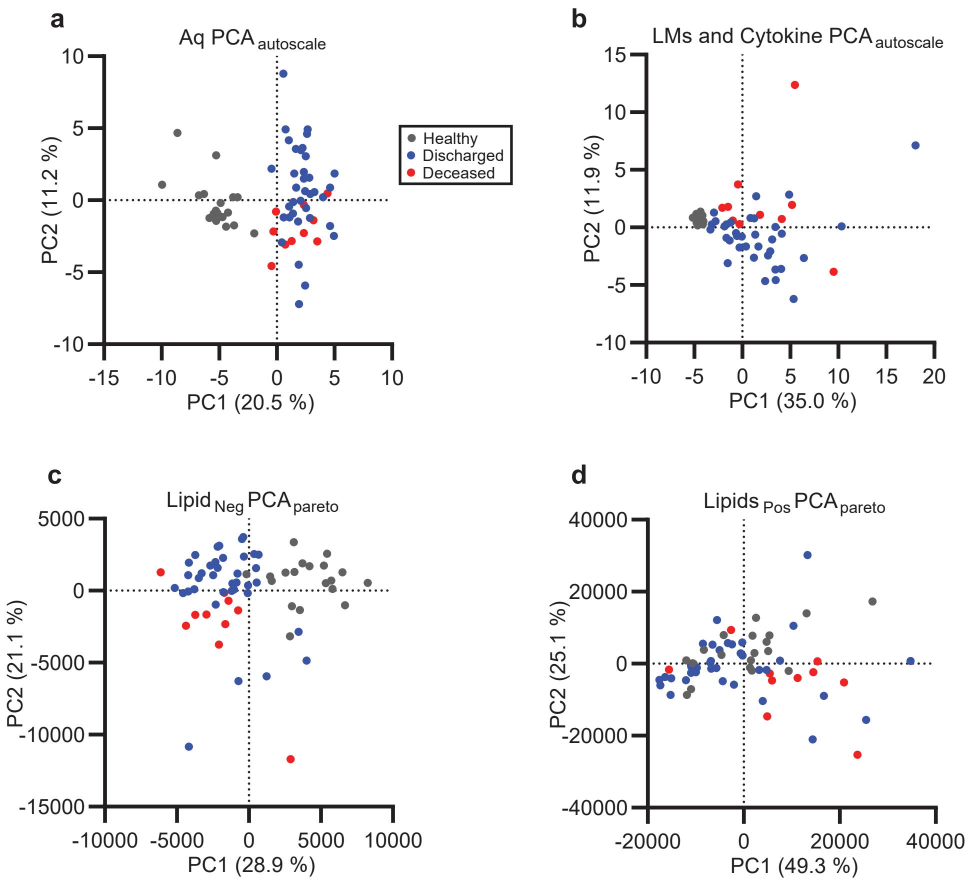

Supplement: Supplement [file Schwarzet.al.eicosanoidsinCOVID19SupplementaryMaterial.pdf]
